# Time course of learning sequence representations in action imagery practice

**DOI:** 10.1016/j.humov.2022.103050

**Published:** 2022-12-20

**Authors:** Stephan F. Dahm, Martina Rieger

**Affiliations:** a Institute of Psychology, Department of Psychology and Sports Medicine, UMIT TIROL - Private University for Health Sciences and Health Technology, Hall in Tyrol, Austria; b Department of Psychology, Faculty of Psychology and Sports Sciences, University of Innsbruck, Austria

**Keywords:** Action imagery practice, Motor imagery practice, Sequence learning, Intermanual transfer, Serial reaction time task

## Abstract

Action imagery practice (AIP) is effective to improve motor performance in a variety of tasks, though it is often less effective than action execution practice (AEP). In sequence learning, AIP and AEP result in the acquisition of effector-independent representations. However, it is unresolved whether effector-dependent representations can be acquired in AIP. In the present study, we investigated the acquisition of effector-independent representations and effector-dependent representations in AEP and AIP in an implicit sequence learning task (a visual serial-reaction-time task, involving a twelve-element sequence). Participants performed six sessions, each starting with tests. A practice sequence, a mirror sequence, and a different sequence were tested with the practice and transfer hand. In the first four sessions, after the tests, two groups performed either AIP (*N* = 50) or AEP (*N* = 54). Improvement in the different sequence indicated sequence-unspecific learning in both AEP and AIP. Importantly, reaction times of the practice hand became shorter in the practice sequence than in the other sequences, indicating implicit sequence learning in both, AEP and AIP. This effect was stronger in the practice hand than in the transfer hand, indicating effector-dependent sequence representations in both AEP and AIP. However, effector-dependent sequence representations were stronger in AEP than in AIP. No significant differences between groups were observed in the transfer hand, although effector-independent sequence representations were observed in AEP only. In conclusion, AIP promotes not only sequence-unspecific stimulus-response coupling and anticipations of the subsequent stimuli, but also anticipations of the subsequent responses.

## 1 Introduction

Action imagery practice (AIP) refers to the repeated use of action imagery with the aim to improve performance (Jeannerod, 1995). AIP has been shown to improve subsequent motor performance, although to a lesser degree as action execution practice (AEP) (Driskell, Copper, & Moran, 1994; Toth, McNeill, Hayes, Moran, & Campbell, 2020). The acquisition of action representations may therefore differ between AIP and AEP, particularly because actual action effects are not available in AIP. The present study investigates the acquisition of effector-dependent and effector-independent sequence representations in AIP and AEP in implicit sequence learning using a serial reaction time task.

AIP has been used in a wide range of tasks and has been shown to improve, for instance, movement speed (arm pointing: Gentili, Papaxanthis, & Pozzo, 2006), error rates (piano playing: Bernardi, De Buglio, Trimarchi, Chielli, & Bricolo, 2013), muscle strength (isometric muscle contraction: Reiser, Büsch, & Munzert, 2011), and movement variability (piano playing: Bernardi et al., 2013). Learning in AIP is assumed to strengthen synaptic efficiency of visual-spatial action effects at the cortical and spinal level (Ruffino, Papaxanthis, & Lebon, 2017) and the recruitment of motor units at a neuro-muscular level (Reiser et al., 2011). A common assumption to explain performance improvements in AIP is that processes similar to AEP take place and that consequently similar associations are learned during AIP and AEP (Jeannerod, 1994). This assumption of functional equivalence is supported by studies demonstrating that the brain areas involved in AIP and AEP largely overlap (Lorey et al., 2013; Sobierajewicz, Przekoracka-Krawczyk, Jaśkowski, Verwey, & van der Lubbe, 2017), that changes in brain activation due to AIP and AEP show similar synaptic efficacy (Ladda, Lebon, & Lotze, 2021; Pascual-Leone et al., 1995; Ruffino et al., 2017), that imagination durations and execution durations are similarly influenced by movement constraints (Dahm & Rieger, 2016a, 2016b; Guillot, Hoyek, Louis, & Collet, 2012), and that the occurrence of imagination errors and execution errors is similarly influenced by motor expertise (Dahm & Rieger, 2019a, 2019b).

From a computational viewpoint, internal models are essential for motor control and motor learning in AEP (Davidson & Wolpert, 2005). In AIP, this may be similar. When executing or imagining an action, motor commands are selected by inverse models. On the basis of the selected motor commands, an efference copy is built which is then used to predict the action outcomes (Wolpert, Diedrichsen, & Flanagan, 2011; Wolpert & Flanagan, 2001), irrespective of whether the action is executed or imagined (Dahm & Rieger, 2019a, 2019b; Rieger, Martinez, & Wenke, 2011). In case the predicted action outcomes deviate from the intended action outcomes, inverse models may adjust the corresponding motor commands (Davidson & Wolpert, 2005). By this, motor learning due to an optimization of the internal models (Thoroughman & Shadmehr, 2000) may occur during AIP similar to AEP. However, the mechanisms in AIP and AEP partly differ: it is necessary that actual activation of the effectors is inhibited in AIP and, consequently, actual action outcomes do not occur in AIP (Guillot, Di Rienzo, Macintyre, Moran, & Collet, 2012; Rieger, Dahm, & Koch, 2017).

Therefore, the acquired representation types may differ between AIP and AEP (Dahm, Weigelt, & Rieger, 2022). Indeed, partly different representations have been observed after AIP than after AEP in darts (Kraeutner, McArthur, Kraeutner, Westwood, & Boe, 2020), golf (Frank, Land, Popp, & Schack, 2014), a tracking task (Kohl & Roenker, 1989), and sequence learning tasks (Amemiya, Ishizu, Ayabe, & Kojima, 2010; Land et al., 2016; Nyberg, Eriksson, Larsson, & Marklund, 2006; Wohldmann, Healy, & Bourne Jr., 2008). In sum, effector-dependent representations are acquired in AEP, but not (or less) in AIP (Amemiya et al., 2010; Dahm et al., 2022; Kraeutner et al., 2020; Sobierajewicz, Jaśkowski, & Van der Lubbe, 2019; Sobierajewicz, Przekoracka-Krawczyk, Jaśkowski, Verwey and van der Lubbe, 2017; Wohldmann et al., 2008). At the same time, effector-independent representations are acquired to the same extend in AEP and AIP (Dahm et al., 2022; Sobierajewicz, Przekoracka-Krawczyk, Jaśkowski, & van der Lubbe, 2017; Wohldmann et al., 2008). Therefore, it has been claimed that action representations are more flexible, i.e., that transfer to other tasks is easier, after AIP than after AEP (Wohldmann et al., 2008). It has been speculated that the differences in acquired action representations between AIP and AEP results from the lack of actual sensory reafferences in AIP (Dahm et al., 2022). Alternatively, inhibitory mechanisms (Guillot, Di Rienzo, et al., 2012; Rieger et al., 2017) may prevent the acquisition of effector-dependent representations.

One way to investigate the types of action representations is to use intermanual transfer tests. Intermanual transfer refers to performance improvements in the transfer hand after unimanual practice with the other hand (Shea, Kovacs, & Panzer, 2011). By comparing performance of the practice and the transfer hand performing spatially same or mirror actions, conclusions about specific types of action representations can be drawn (Dahm et al., 2022). *Effector-dependent* representations involve motor commands that are restricted to the practiced effector (Imamizu & Shimojo, 1995; Panzer, Krueger, Muehlbauer, Kovacs, & Shea, 2009). Using the intermanual transfer paradigm, they are observable in better performance in the practice hand than in the transfer hand. *Effector-independent intrinsic* representations imply body-based motor coordinates restricted to homologous effectors (Criscimagna-Hemminger, Donchin, Gazzaniga, & Shadmehr, 2003). This results in homologous intermanual transfer, indicated by better performance in mirror actions in the transfer hand than in control actions in the transfer hand. *Effector-independent visual-spatial extrinsic* representations imply environment-based coordinates not restricted to effectors (Imamizu & Shimojo, 1995). This results in visual-spatial intermanual transfer, indicated by better performance in spatially equal actions in the transfer hand than in control actions in the transfer hand.

Because the acquisition of effector-dependent representations in AIP may depend on the type of task, we investigated what types of representations are acquired in AIP using a serial reaction time task. In AIP learning effects have already been shown using the serial reaction time task (Kraeutner, MacKenzie, Westwood, & Boe, 2016; Shanks & Cameron, 2000; Wohldmann et al., 2008). In comparison to previous investigations on the representation types of explicit sequences (Dahm et al., 2022; Sobierajewicz, Przekoracka-Krawczyk, Jaśkowski, & van der Lubbe, 2017), the serial reaction time task involves implicit sequence learning. In the present study, participants reacted to a series of visual stimuli with visual-spatially corresponding keypresses. Unknown to participants, the keypresses followed a particular sequence. To enforce implicit sequence learning (instead of explicit sequence learning), we used a relatively long sequence of twelve elements which started randomly at any point of the sequence. We expected to replicate and extend findings on implicit sequence learning in AIP (Kraeutner et al., 2016; Shanks & Cameron, 2000; Wohldmann et al., 2008). Therefore, we combined investigations on learning in the transfer hand and in the practice hand (Sobierajewicz, Przekoracka-Krawczyk, Jaśkowski, & van der Lubbe, 2017; Wohldmann et al., 2008), the direction of intermanual transfer (Criscimagna-Hemminger et al., 2003), transfer to the practice sequence, a mirror sequence, and a different sequence (Panzer et al., 2009), and the course of learning (Panzer et al., 2009) in one experiment.

Previous studies have already investigated implicit sequence learning in AIP (Kraeutner et al., 2016; Shanks & Cameron, 2000; Wohldmann et al., 2008). However, the representation types have not been distinguished as rigorously as explained above. For instance, it has been shown that implicit sequence learning in the practice hand is stronger in AIP than in control practice, but less than in AEP (Shanks & Cameron, 2000). Here, tests in the transfer hand were not performed. Further, in the transfer hand, implicit sequence learning was observed to be similar in AIP and AEP (Kraeutner et al., 2016), or even superior in AIP than in AEP (Wohldmann et al., 2008). Based on these results, it was suggested that effector-independent representations of a motor sequence are acquired equally well in AIP and AEP (Kraeutner et al., 2016; Wohldmann et al., 2008). Nevertheless, direct comparisons between transfer and practice hand were not performed to isolate the acquisition of effector-dependent representations.

Effector-independent representations are assumed to be acquired similarly in AIP and AEP because transfer to the unpracticed hand has been observed after AIP and AEP (Kraeutner et al., 2016; Sobierajewicz, Przekoracka-Krawczyk, Jaśkowski, & van der Lubbe, 2017; Wohldmann et al., 2008). However, effector-independent representations can be further subdivided into intrinsic and visual- spatial extrinsic effector-independent representations which may not evolve at the same learning stage in AIP and AEP. It has been shown that in AEP, intrinsic effector-independent representations evolve at later stages of learning than visual-spatial extrinsic effector-independent representations (Bapi, Doya, & Harner, 2000; Panzer et al., 2009). In AIP, this has not been shown yet. We expected that, due to similar mechanisms in AIP and AEP, intrinsic effector-independent representations evolve at later stages of learning than visual-spatial extrinsic effector-independent representations. Alternatively, intrinsic effector-independent representations may not evolve at all in AIP and AEP if the task is primarily visual-spatial in nature (Dahm et al., 2022).

In addition, we investigated the direction of intermanual transfer. Intermanual transfer can be asymmetric, i.e., it occurs more strongly from the left to the right hand or vice versa. For instance, in a writing task, transfer was observed from the right to the left hand but not in the other direction (Lohse, Healy, & Sherwood, 2010). Intermanual transfer can also be symmetric, i.e., it occurs in both directions similarly. It has been proposed that whether the acquired representations are available for both limbs depends on characteristics of the task (Criscimagna-Hemminger et al., 2003) and the type of outcome measure (Pan & Van Gemmert, 2013). In the serial reaction time task, symmetric transfer was observed in AEP (Chase & Seidler, 2008). Therefore, we expected transfer in both directions in AEP. Further, we expected the same transfer direction(s) in AIP (Wohldmann et al., 2008).

Further, we investigated participants’ knowledge of the sequence. We did not expect participants to be able to freely generate the practice sequence due to its implicit nature during acquisition. However, participants may recognize the sequence as this is not only based on knowledge but may also indicate an unspecific intuition of familiarity during execution of the test. Most importantly, we did not expect free generation or recognition performance to differ after AEP and AIP (Dahm et al., 2022).

Recapitulating the principal hypotheses, we expected effector-independent visual-spatial representations at early stages of both AEP and AIP. Effector-independent intrinsic representations and effector-dependent representations were expected at late stages of learning in AEP and to be weaker after AIP than after AEP. Further, we expected robust learning effects which should evolve in stable representations after four weeks without further practice after both AEP and AIP (Dahm et al., 2022).

## 2 Methods

### 2.1 Participants

Originally, 97 participants were tested. Nine participants had technical problems which resulted in partially missing data. The remaining 88 participants reported to have at least moderately clear and vivid action imagery as assessed by the German Version (Dahm, Bart, Pithan, & Rieger, 2019) of the Vividness of Movement Imagery Questionnaire (Roberts, Callow, Hardy, Markland, & Bringer, 2008). Two participants of the AIP group and two participants of the AEP group were excluded due to very slow performance in the first pretest (RT >3 standard deviations above the mean). Of the remaining 84 participants, the distribution of sex as well as means and standard deviations of age, the laterality index (Oldfield, 1971), and the ratings on the factors of the vividness of movement imagery questionnaire (external visual imagery, internal visual imagery, and kinesthetic imagery, Dahm et al., 2019; Roberts et al., 2008) are shown separately for both groups in Table 1. All participants gave informed consent. All procedures performed in the present study were in accordance with the 1964 Helsinki declaration and its later amendments. The experiment was approved by the local ethics committee. Participants performed the experiment for course credit.

The required sample size for an interaction between four groups (the combination of practice group and practice hand) and six test sessions was estimated with G*Power (Faul, Erdfelder, Lang, & Buchner, 2007). We assumed an effect size of *f* = .25 and correlations among repeated measures of *r* = .4. Alpha was set at .05 and the power (1-beta) at .8 which resulted in a minimum sample size of *N* = 92 (*n* = 23 per group).

### 2.2 Materials and procedure

The experiment was run on participants’ personal notebooks using OpenSesame 3.1.4 (Mathôt, Schreij, & Theeuwes, 2012). The experiment file including all stimuli and instructions is available at https://osf.io/brd72.

Participants performed a four-choice serial reaction time task using fingers of the same hand (adapted from Reber & Squire, 1998). Visual stimuli consisted of four horizontally aligned boxes (2 cm × 3 cm) with an asterisk (diameter: 1 cm) in one of the boxes. Participants responded to each visual stimulus by pressing a spatially corresponding response key as fast as possible. As response keys, the keys ‘F’, ‘G’, ‘H’, and ‘J’ of the computer keyboard were pressed with the index, middle, ring, and little finger, respectively. Participants were not informed that the responses followed a particular sequence.

Four sequences were used. Each sequence consisted of twelve elements: Sequence A (GHGJFHFJHJGF), Sequence A mirrored (HGHFJGJFGFHJ), Sequence B (JHGJGHFGFJFH), and Sequence B mirrored (FGHFHGJHJFJG). In all sequences, each element appeared equally often, the same element was not repeated on successive trials, and each transition between elements occurred equally often. Thus, first order learning was not possible (Reber & Squire, 1998).

**Fig. 1:**
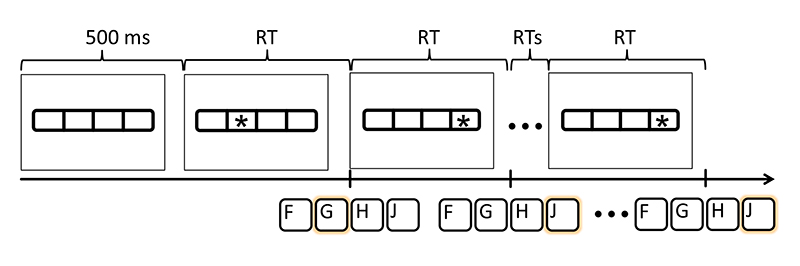
Depiction of stimuli and responses. A block started with four empty boxes. After 500 ms an asterisk appeared in one of the boxes. Immediately after a response, the asterisk appeared in another box. Reaction time (RT) was recorded for each response.

A block of sequence elements started always with four empty boxes on the screen (no asterisk). After 500 ms, the first stimulus of the sequence appeared. Within a sequence the starting stimulus was random. The task was self-paced and both, correct and incorrect responses, triggered the presentation of the next stimulus (Fig. 1).

Participants completed six sessions (Table 2). To ensure that participants followed the instructions and to answer any questions, Session 1, Session 5, and Session 6 were completed in a laboratory with an experimenter present. Sessions 2–4 were completed at home, with participants sitting at a table in a quiet room. Practice sessions were at least two days apart, on average 3.2 days (*SD* = 0.3 days).

All sessions started with a *pretest*. In the pretest, sequence blocks consisted of four repetitions of a sequence resulting in 48 consecutive responses. Each sequence (A, AM, B, and BM) was performed with each hand, resulting in eight blocks. The order of the hands was blocked and counterbalanced across participants. The order of the sequences was random, but equal for each hand.

In the first four sessions, testing was followed by six practice blocks. Practice blocks consisted of 10 sequence repetitions resulting in 120 consecutive responses. Participants were randomly assigned into four groups. They practiced either with the left or right hand and performed either AIP or AEP. In *AEP*, they were asked to press the keys with the corresponding finger of the practice hand. In *AIP*, participants were asked to imagine pressing the keys with the corresponding finger of the practice hand. Simultaneously with the actual or imagined keypresses, both groups pressed the shift key with the thumb of the other hand which triggered the end of the stimulus during practice. Using this procedure, we primarily aimed to increase participants’ commitment to perform imagery during practice, because they were aware that we were tracking their timing during imagery. The practice sequence was counterbalanced across participants. Each practice phase took about 10–20 min as suggested for AIP (Driskell et al., 1994; Toth et al., 2020).

After the last practice block in Session 4, participants reported their kinesthetic, visual, and auditory representation of the (imagined) action on a rating scale (see supplemental material). After each practice session, participants performed a posttest^1^ which was the same as the pretest for each participant.

Session 5 was performed on average 4.5 days (*SD* = 1.5 days) after the last practice session. After the test in Session 5, a *free generation* test was performed. Participants were asked to execute 13 keypresses in the order of the practice sequence with the practice hand while empty boxes, but no stimuli, were presented on the screen. This was followed by a *recognition* test. In the recognition test, each sequence was presented the same way as during the test blocks. Blocks consisted of 13 elements (i.e., the first element of the sequence was repeated at the end). After each sequence, participants rated whether the performed sequence corresponded to the practice sequence on a rating scale ranging from 1 – “very unlikely” to 9 – “very likely”. The order of the four sequences in the recognition test was randomized.

In Session 6, participants performed the pretest only. Session 6 was conducted on average 28.3 days (*SD* = 3. 6 days) after Session 5.

### 2.3 Data analysis

Reaction time (RT) was defined as the time between presentation of a stimulus and its corresponding response. RTs of the first twelve responses in each sequence were not included into analysis. RTs of an erroneous response (r) and its subsequent response (r + 1) were not included into analysis. Error rates were below 10% (see supplemental material). Of the remaining responses, median RTs were calculated for each condition. To obtain equal reliability across practice, mirror and different sequences, only one of the two different sequences was randomly chosen.

In a first step, we analyzed RTs in the pretests. In this analysis, we checked whether transfer differed between the groups that trained with the right and the left hand, and we looked at sequence-unspecific effects (i.e., on effects in RTs that did not interact with sequence effects) and sequence-specific effects (i.e., on differences in RTs between sequences). In a second step, to compare the extent of sequence-specific effects between groups and hands, we calculated and analyzed a sequence learning index by subtracting RTs of the practice sequence from RTs of the different sequence (see Kraeutner et al., 2016). In a third step, we analyzed free generation and recognition performance. To analyze free generation performance, we calculated the number of triplets in the free generation test that were compatible with the practice sequence and the mirror sequence. This indicates the amount of explicit knowledge of the sequence structure (Bird & Heyes, 2005). Recognition performance was analyzed using the ratings of the recognition test.

Dependent variables were analyzed using mixed model ANOVAs. If Mauchly’s test indicated that the assumption of sphericity was violated, we report Huyn-Feld corrected degrees of freedom and *p*-values. Further comparisons were conducted using *t*-tests with Sidak-adjusted pairwise comparisons. In case that more than one comparison was non-significant, we report minimum p-values (*p*_min_). In case that more than one comparison was significant, we report maximum p-values (*p*_max_). Statistical significance was set at *p* < .05.

Additional analyses of RTs and strength of representation during practice are shown in the supplemental material. Raw data as well as the syntax for data preparation and data analyses are available at https://osf.io/brd72.

## 3 Results

### 3.1 Reaction times in the pretests

Mean RTs and standard errors are presented in the supplement. A repeated measures ANOVA with the between-factors Practice group (AIP, AEP) and Practice hand (left, right) and the within-factors Test hand (practice, transfer), Sequence (practice, mirror, different), and Session (1, 2, 3, 4, 5, 6) was performed on RTs. Statistical parameters (*F*, *df*, *p*, $\eta_{p}^{2}$) of the ANOVA are shown in Appendix A.

**Fig. 2:**
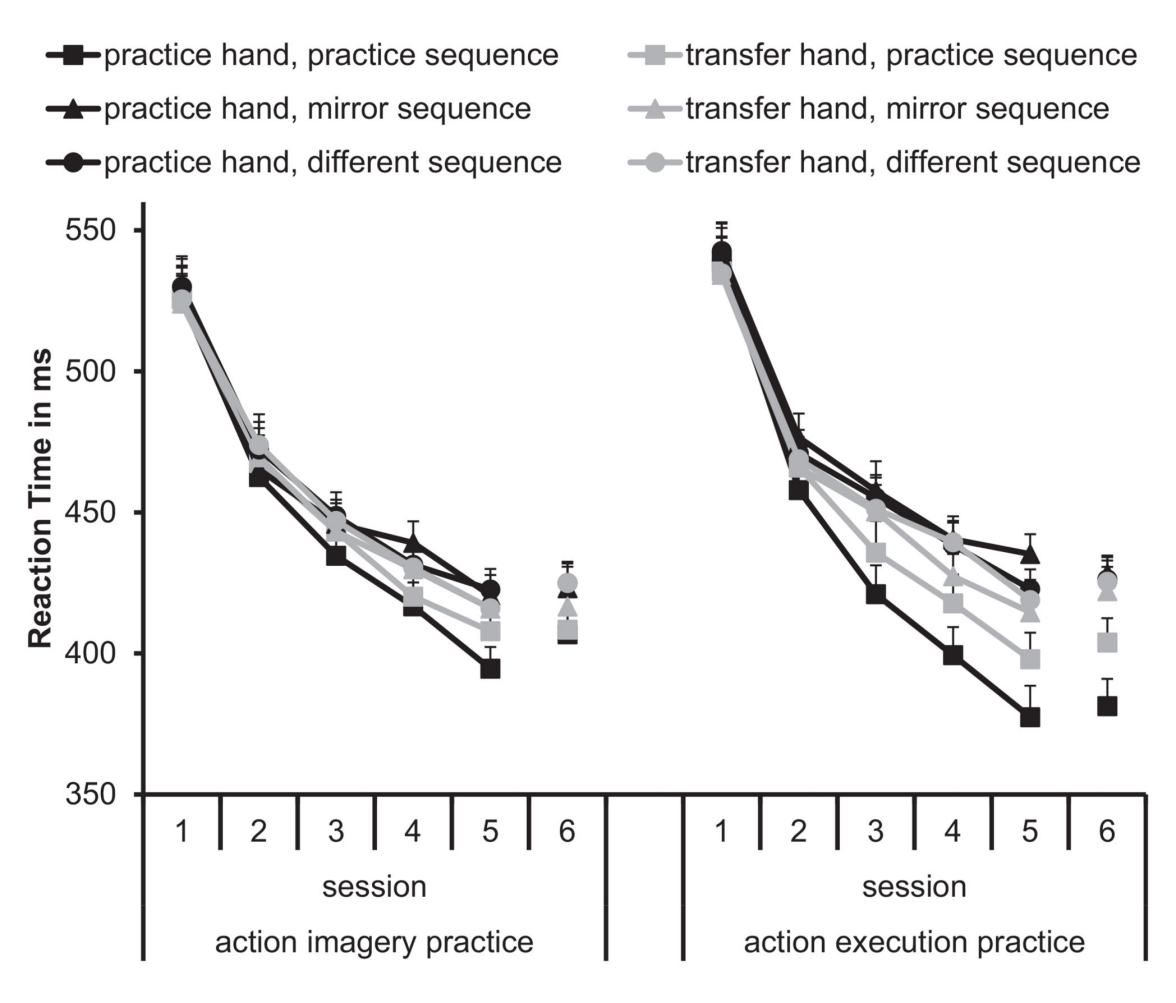
Means and standard errors of reaction times depending on hand (practice, transfer), sequence (practice, mirror, different), and session (1, 2, 3, 4, 5, 6) separately for the action imagery practice group and the action execution practice group.

A significant interaction between *Practice hand and Test hand*, revealed that RTs were significantly shorter in the right hand (practice hand for the right-hand-practice group: *M* = 444 ms, transfer hand for the left-hand-practice group: *M* = 448 ms) than in the left hand (practice hand for the left-hand-practice group: *M* = 456 ms, transfer hand for the right-hand-practice group: *M* = 451 ms). Further, the interaction between Practice hand, Practice group and Session reached significance. However, posthoc-comparisons between the practice hand groups were not significant (*p*_min_ = .059). All other comparisons with the factor Practice hand were not significant (maximum $\eta_{\text{p}}^{2}=.02$). Importantly, the factor Practice hand did not significantly interact with the factors Sequence and Practice group. To clearly present the important data, we decided to average the data over the factor Practice hand for Fig. 2, which shows means and standard errors of RTs.

The significant *main effect Session* indicated a significant improvement from session to session (*M*_Session1_ = 532 ms, *M*_Session2_ = 468 ms, *M*_Session3_ = 444 ms, *M*_Session4_ = 428 ms, *p*_max_ < .001), except from Session 5 (*M* = 412 ms) to Session 6 (*M* = 415 ms, *p* = .989). The significant main effect Sequence was modified by significant interactions between a) Practice group and Sequence, b) Test hand and Sequence, c) Session and Sequence, d) Practice group, Test hand, and Sequence, and e) Practice group, Session, and Sequence.

The significant interaction between Sequence, Practice group, and Session indicated the following: The mirror sequence did not significantly differ from the different sequence in any session in both groups (*p*_max_ = .24). In AEP, RTs in the practice sequence were significantly shorter than in the different sequence from Test 3 onwards (*p*_max_ < .001). In AIP, RTs in the practice sequence were significantly shorter than in the different sequence in Test 5 (*p* = .002) and Test 6 (*p* = .002). Hence, sequence learning occurred earlier in AEP than in AIP.

The significant interaction between Sequence, Practice group, and Test hand indicated the following: The mirror sequence did not significantly differ from the different sequence in any hand in both groups (*p*_max_ = .191). In AEP, RTs in the practice sequence were significantly shorter than in the different sequence in both, the practice hand (*p* < .001) and the transfer hand (*p* < .001). In AIP, RTs in the practice sequence were significantly shorter than in the different sequence in the practice hand (*p* < .001), but not in the transfer hand (*p* = .136).

To further investigate the effects of the two interactions, we performed the theoretically meaningful comparisons between the practice sequence and the different sequence (for each Session, Test hand, and Practice group).

#### Practice sequence in the practice hand

In AEP, RTs in the practice sequence were significantly shorter than in the different sequence from Session 3 onwards (*p*max < .0014). This difference was significantly lower in Session 1 and Session 2 than in Session 4 and the following sessions (*p*_max_ = .003). In AIP, RTs in the practice sequence were significantly shorter than in the different sequence from Session 4 onwards (*p*_max_ = .032).

#### Practice sequence in the transfer hand

In AEP, RTs in the practice sequence were significantly shorter than in the different sequence from Session 3 onwards (*p*_max_ = .005). In AIP, RTs did not significantly differ between the practice sequence and the different sequence (*p*_min_ = .074).

In sum, we found that sequence learning occurred in the practice sequence in the practice hand, both in AIP and AEP and that sequence learning occurred in the practice sequence in the transfer hand in AEP. No systematic effects were observed in the mirror sequence. To analyze the amount of sequence-specific learning (e.g., between sessions, tests hands, and practice groups), we analyzed the sequence learning index which is detailed in the next section.

### 3.2 Sequence learning index

**Fig. 3:**
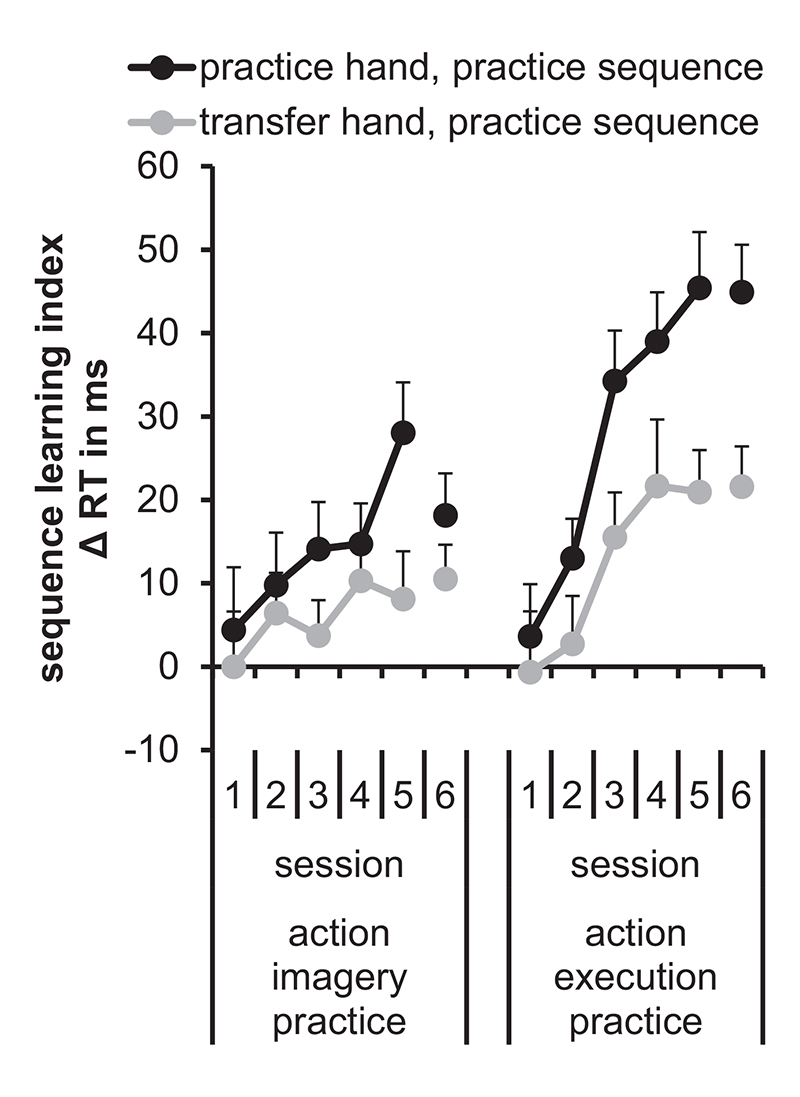
Means and standard errors of the sequence learning index depending on hand (practice, transfer), and session (1, 2, 3, 4, 5, 6) separately for the action imagery practice group and action execution practice group. Note that we did not calculate the sequence learning index for the mirror sequence, because no significant differences were observed between the mirror sequence and the different sequence.

To focus on the extent of sequence-specific learning, the sequence learning index (see Kraeutner et al., 2016 for a similar procedure) was calculated for the practice sequence by subtracting the RTs of the practice sequence from the RTs of the different sequence. Means and standard errors of the sequence learning index are shown in Fig. 3. A repeated measures ANOVA with the between factor Practice group (AIP, AEP) and the within factors Hand (practice, transfer), and Session (1, 2, 3, 4, 5, 6) was performed on the sequence learning index.

The significant main effect Session, *F* (4.1, 339.7) = 11.5, *p* < .001, $\eta_{\text{p}}^{2}=.12,$ indicated that the sequence learning index was significantly lower in Session 1 than in Session 3 and the following sessions (*p*_max_ = .02). Further, it was lower in Session 2 than in Session 4 and the following sessions (*p*_max_ = .006). Session 3 to Session 6 did not significantly differ between each other (*p*_min_ = .166). Hence, sequence-specific learning increased in the first sessions and remained stable after a month without further practice.

The significant main effect Practice group, *F*(1, 83) = 7.7, *p* = .007, $\eta_{\text{p}}^{2}=.09,$ was modified by the significant interaction between Practice group and Session, *F* (4.1, 339.7) = 2.7, *p* = .032, $\eta_{\text{p}}^{2}=.03.$ The sequence learning index did not significantly differ between AEP and AIP in Session 1 and Session 2 (*p*_min_ = .926). From Session 3 onwards, it was significantly higher in AEP than in AIP (*p*_max_ = .033). Hence, sequence-specific learning was stronger in AEP than in AIP.

The significant main effect Hand, *F* (1, 83) = 29.4, *p* < .001, $\eta_{\text{p}}^{2}=.26,$ indicated that the sequence learning index was significantly higher in the practice hand (*M* = 22 ms) than in the transfer hand (*M* = 10 ms). This indicates effector-dependent representations in both AEP and AIP.

All remaining interactions were not significant: Practice group x Hand: *F* (1, 83) = 3.1, *p* = .082, $\eta_{\text{p}}^{2}=.04;$ Hand x Session: *F* (4.7, 387.5) = 1.6, *p* = .153, $\eta_{\text{p}}^{2}=.02;$ Practice group x Hand x Session: *F* (4.7, 387.5) = 0.4, $\eta_{\text{p}}^{2}<.01.$

### 3.3 Free generation and recognition performance

**Fig. 4:**
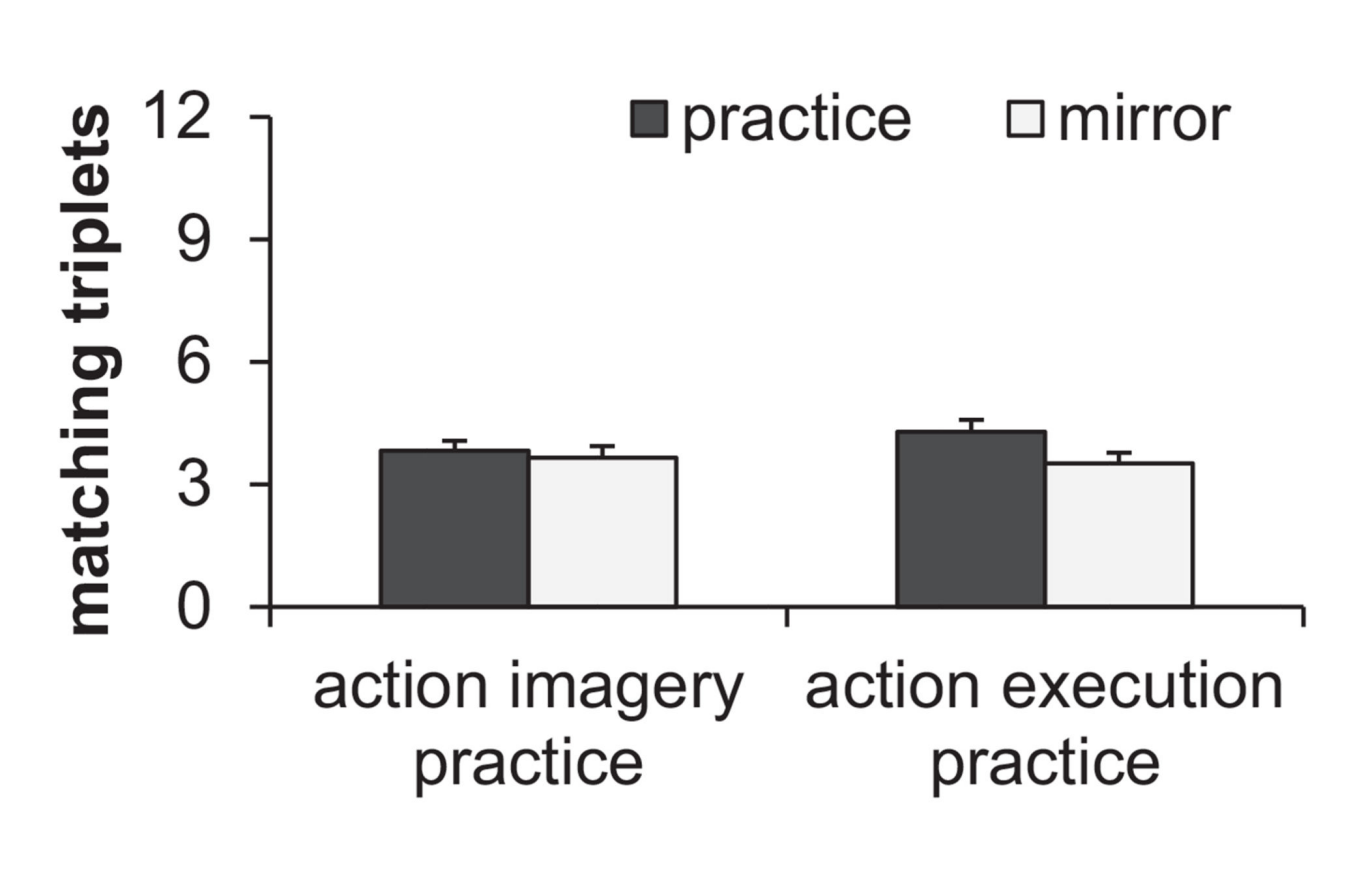
Means and standard errors of the free generated triplets matching with the practice sequence and mirror sequence separately for the practice groups.

To analyze the free generation performance a repeated measures ANOVA with the between factor Practice group (AIP, AEP) and the within factor Sequence (practice, mirror) was calculated on matching triplets. Means and standard deviations are shown in Fig. 4. All effects were not significant (Practice group: *F* < 1; Sequence: *F* (1, 83) = 2.6, *p* = .112, $\eta_{\text{p}}^{2}=.03;$ Practice group x Sequence: *F* (1, 83) = 1, *p* = .313, $\eta_{\text{p}}^{2}=.01$).

**Fig. 5:**
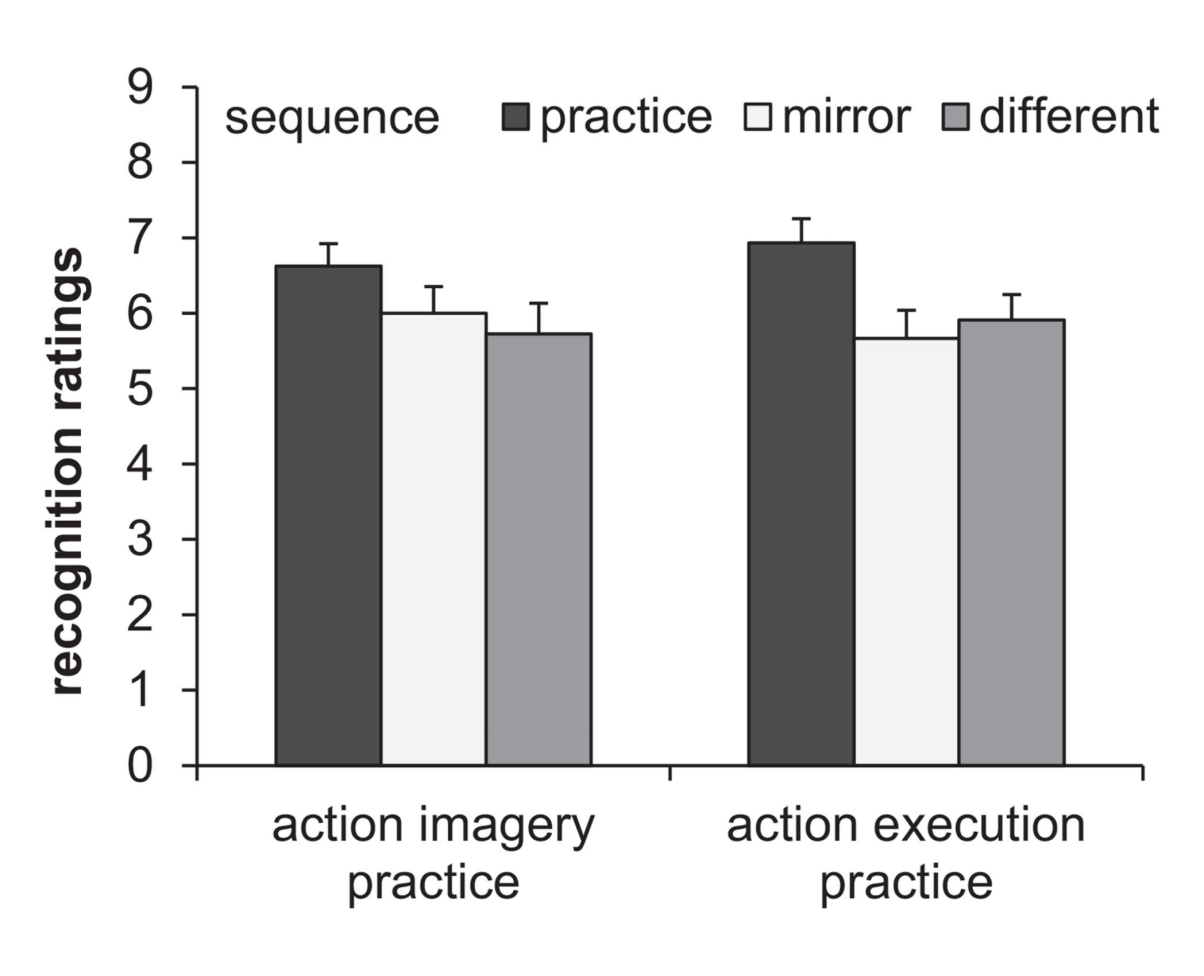
Means and standard errors of the recognition ratings of the sequences (practice, mirror, different) separately for the action imagery practice group and action execution practice group.

To analyze the recognition performance a repeated measures ANOVA with the between factor Practice group (AIP, AEP) and the within factor Sequence (practice, mirror, different) was calculated on the recognition ratings. Means and standard errors are shown in Fig. 5. The significant main effect of Sequence, *F* (2, 83) = 6.2, *p* = .002, $\eta_{\text{p}}^{2}=.07,$ indicated significantly higher ratings for the practice sequence (*M* = 6.8, *SE* = 2) than for the mirror sequence (*M* = 5.8, *SD* = 2.4) and the different sequence (*M* = 5.8, *SD* = 2.4). The main effect Practice group and the interaction between Practice group and Sequence were not significant, both *F* < 1.

## 4 Discussion

Are effector-dependent representations acquired with AIP? Do they appear later in AIP than in AEP? Do effector-independent (visual-spatial or intrinsic) representations differ after AIP and AEP? To answer these questions, we investigated implicit sequence learning using the serial reaction time task in AIP and AEP. We investigated the time course of learning in the practice and transfer hand. A general sequence-unspecific improvement was observed from Session 1 to Session 5 in both practice groups and both hands. In the course of learning, RTs in the practice sequence became shorter than in the different sequence in both practice groups. The sequence learning indicated that such sequence-specific learning effects were stronger in AEP than in AIP. In both, AEP and AIP sequence-specific learning was larger in the practice hand than in the transfer hand indicating effector-dependent representations. After practice, free generation and recognition performance of the practice sequence did not differ significantly between AEP and AIP.

### 4.1 Sequence-unspecific learning

In all sequences, RTs became shorter after each practice session. Such sequence-unspecific improvements were observed in both practice groups and in both hands. Sequence-unspecific learning may have been caused by general adaptations to the task (Kraeutner et al., 2016). For instance, participants may have optimized stimulus processing and strengthened the associations between stimuli and their corresponding responses in the course of learning (Verwey, Shea, & Wright, 2015). Alternatively, because the same test was used several times during the experiment, sequence-unspecific performance improvements may be caused by repeated testing (Butler, 2010). Although no further improvement occurred, sequence-unspecific learning effects remained stable, as they were maintained after four weeks without practice in Session 6.

### 4.2 Sequence-specific learning

In the practice hand, lower RTs in the practice sequence than in the different sequence indicated sequence-specific learning. This was observed earlier in AEP (from Session 3 onwards) than in AIP (in Session 5 and Session 6). Further, sequence learning was stronger in AEP than in AIP from Session 3 onwards. Hence, sequence learning was faster and stronger in AEP than in AIP. Such findings are in line with previous results showing generally higher learning effects after AEP than after AIP (Driskell et al., 1994; Toth et al., 2020), though most studies did not distinguish between different types of representations.

#### 4.2.1 Effector-dependent representations of the sequence

Sequence learning was stronger in the practice hand than in the transfer hand indicating effector-dependent representations in both AEP and AIP. The fact that effector-dependent representations were observed in AIP is particularly interesting, because this was not always observed in previous studies (Dahm et al., 2022; Land et al., 2016).

**Fig. 6:**
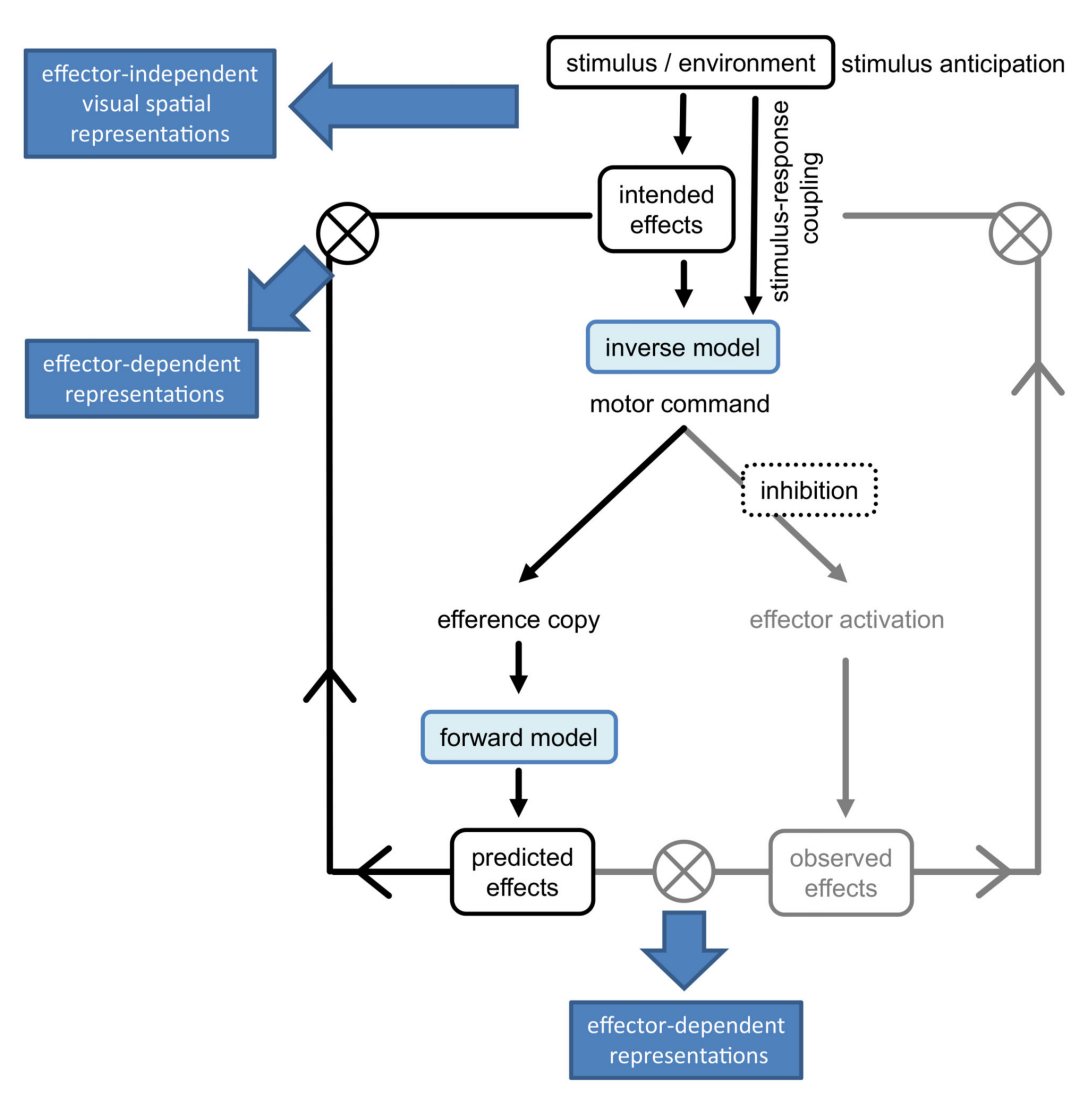
Processes and acquisition mechanisms for effector-independent and effector-dependent representations in action execution practice (adapted from Blakemore, Wolpert, & Frith, 2002; Dahm & Rieger, 2019a). The processes and acquisition mechanisms in grey do not occur in action imagery practice.

The present data indicate that AIP can activate motor representations directly, most likely due to a motor simulation evoking effector-dependent representations (Ingram, Kraeutner, Solomon, Westwood, & Boe, 2016). Similarly, effector-dependent representations have been observed after action observation practice (AOP; Bird & Heyes, 2005). AOP differs from AIP (Kim, Frank, & Schack, 2017), but actual sensory motor reafferences based on action feedback are not involved in both. Such reafferences are assumed to enhance effector-dependent representations in AEP (Kraeutner et al., 2020). Hence, AOP and AIP may promote the development of effector-dependent representations in another way. Most likely, the action is simulated using inverse and forward models (Blakemore & Decety, 2001) predicting the action consequences that include sensory motor feedback (Fig. 6). Hence, AIP involves a full-fledged action simulation including motor components (Davidson & Wolpert, 2005; Grush, 2004; Ingram et al., 2016).

Possibly, the acquired effector-dependent representations allow the learner to anticipate the subsequent responses which can thereby be prepared already in advance to the appearance of the corresponding stimulus. To speculate, effector-dependent representations may be acquired in AIP only when practicing implicitly (Ingram et al., 2016), but not when practicing explicitly (Dahm et al., 2022; Land et al., 2016). Explicit thinking and reasoning strategies (Liao & Masters, 2001) may interfere with learning effectordependent representations in AIP in explicit learning tasks. Additionally, in previous studies all sequence elements were available when starting (Dahm et al., 2022; Land et al., 2016). Therefore, an anticipation of the subsequent action elements was not necessary. This process may however contribute to performance improvements in AIP in the present study but did not occur in those studies. Instead, effector-dependent representations in these tasks may result from optimized dynamic movement trajectories (Land et al., 2016; Lohse et al., 2010), which are acquired after AEP but not after AIP (Dahm et al., 2022).

#### 4.2.2 Visual-spatial effector-independent representations of the sequence

Sequence learning of the practice sequence in the transfer hand was significantly stronger in AEP than in AIP. Further, in the transfer hand, the difference between practice sequence and different sequence was significant in AEP only, but not in AIP. This stands in contrast to previous studies showing that extrinsic visual-spatial effector-independent representations can be acquired in both types of practice (Dahm et al., 2022; Ingram et al., 2016; Sobierajewicz, Przekoracka-Krawczyk, Jaśkowski, & van der Lubbe, 2017; Wohldmann et al., 2008). Interestingly, these sequence-specific learning effects in the transfer hand remained stable in AEP after six weeks without further practice in Session 6. Most likely, using the serial reaction time task, visual-spatial effector-independent representations are developed using mechanisms that are available in AEP e.g., stimulus anticipation (Koch & Hoffmann, 2000) or stimulus-response (imagined or actual) coupling (Wenderoth & Weigelt, 2009). Mechanisms to predict the action consequences such as forward models may however not be involved in the acquisition of effector-independent representations (Fig. 6).

#### 4.2.3 Intrinsic effector-independent representations of the sequence

We had expected shorter RTs in the mirror sequence than in the different sequence in the transfer hand in later stages of learning, which would have been an indicator of intrinsic effector-independent representations (Panzer et al., 2009). However, this effect was not observed neither in AIP nor in AEP. We therefore conclude that intrinsic effector-independent representations were not acquired in our study. Hence, the acquired effector-dependent representations that helped to anticipate and prepare subsequent responses in the practice hand, did not enhance homologous performance in the transfer hand.

One may argue that four practice sessions involving a total of 240 sequence repetitions is not enough to develop intrinsic effector-independent representations. However, other studies observed intrinsic effector-independent representations already after one practice session of 99 sequence repetitions in AEP (Panzer et al., 2009). More likely, the lack of intrinsic effector-independent representations was due to task characteristics. The present task required a visual-spatial match of stimuli and responses. Symbolic or acoustic (Kraeutner et al., 2016) stimulus material may lead to intrinsic effector-independent representations rather than the visual- spatial stimuli used in the present study.

#### 4.2.4 Sequence knowledge

We further investigated the implicitness (or explicitness) of the sequence representation by using a free generation and a recognition test which were solely executed in the practice hand. We expected that a completely explicit sequence representation would result in nearly perfect free generation and recognition performance. However, this was not observed. Participants were not able to freely generate the practice sequence. The number of freely generated triplets matching with the practice sequence did not significantly differ from the number of triplets matching with the mirror sequence, which serves as a control for guessing (Bird & Heyes, 2005). However, participants were able to distinguish the practice sequence from the different sequence and the mirror sequence in the recognition test. Thus, participants acquired presumably implicit knowledge of the practice sequence. When confronted with the recognition test, participants may have had an undetermined intuition that one of the sequences matched better with the practice sequence without explicitly being aware of the sequence structure (see also Verwey et al., 2015).

Most interestingly for the present study, participants of the AIP group did not significantly differ from those in the AEP group in both tests. This indicates that explicitness of the sequence representation does not differ after AIP and AEP. Therefore, the stronger effector-dependent sequence learning effects in AEP than in AIP were not caused by explicit sequence knowledge.

## 5 Symmetry of intermanual transfer

In the present study, the direction of transfer did not influence the extent of intermanual transfer: transfer to the unpracticed hand did not depend on whether the right or left hand was practiced. Such symmetric intermanual transfer has been observed in drawing a waveform (Panzer et al., 2009), sequential finger movements (Chase & Seidler, 2008; Dahm et al., 2022), speed tapping (Koeneke, Battista, Jancke, & Peters, 2009), and mice tracking (van Mier & Petersen, 2006). Most importantly for the present study, the influence of the direction of transfer did not differ between AEP and AIP.

## 6 Limitations and perspectives

When investigating action imagery and AIP, it is always difficult to ascertain that participants indeed perform imagery, as this is not directly observable (Dahm, 2020). In the present study, participants pressed a different key with a different effector during practice. Imagination durations (analyzed in the supplement) showed a similar decrease in RTs in the course of learning in AIP and AEP, which indicates that participants adhered to the instructions to perform imagery. Furthermore, the observation that evidence for sequencespecific learning was obtained in AIP indicates that participants adhered to the instructions.

One may argue that pressing the additional key during practice presents a confound, because two tasks were performed simul-taneously. In AEP, this may have led to dual-task costs hampering sequence learning (Röttger, Zhao, Gaschler, & Haider, 2021). However, the additional keypress (thumb of the other hand) was a one-choice reaction which was much simpler than the four-choice reaction of the practiced task. We therefore assume that the additional keypress was integrated quickly into the serial reactions and that it was perceived as belonging to the task (Koch, Poljac, Müller, & Kiesel, 2018). In AIP, executing the additional key may have led to a mix of AEP and AIP. Indeed, considering this, AIP was not purely based on imagery only, but involved executable motor commands with the other hand. However, sequence-specific learning effects in AIP cannot be explained with the repetitive responses of the additional key. Still, it could be that execution in the unpracticed hand (= transfer hand) during practice hampered the acquisition of effector-independent intrinsic representations.

Visual-spatial congruency of stimuli and responses in the practice sequence may have led to anticipatory eye-movements. Such learning of eye-movements using the serial reaction time paradigm (Marcus, Karatekin, & Markiewicz, 2006) was possible in both, AEP and AIP, because the eyes were kept open in both conditions. Thus, learning of effector-independent visual-spatial representations may have been supported by executed actions (eye-movements).

## 7 Conclusion

Sequence-unspecific learning as well as sequence-specific learning were observed in AEP and in AIP. Sequence-specific effector-dependent representations were acquired faster and stronger in AEP than in AIP. Effector-dependent representation may develop due to anticipation and preparation of subsequent responses in the practice hand. In the transfer hand, effector-independent representations were acquired in visual-spatial extrinsic coordinates rather than in intrinsic body-based parameters. Effector-independent visual- spatial representations may develop due to anticipation of subsequent stimuli and stimulus-response coupling. Finally, sequence knowledge did not significantly differ between AEP and AIP. We therefore conclude that implicit sequence learning is based on similar mechanisms in AIP and AEP (Grush, 2004; Jeannerod, 2001). Effector-independent visual-spatial representations may evolve due to the repetitive presentation of the stimulus material, which is action independent. Effector-dependent representations however may be optimized due to comparisons of predicted action effects and intended action effects. Because predicted action effects can be compared additionally with actual action effects in AEP, but not in AIP, effector-dependent representations evolve earlier and stronger in AEP than in AIP.

## Supplementary Material

Appendix

## Figures and Tables

**Table 1:** Sociodemographic data of the action imagery practice group and the action execution practice group. To compare the practice groups, a X^2^ Test was calculated for the distribution of sex and t-tests were computed for the remaining variables.

	Action imagery practice	Action execution practice	p
sex, *N*_f_ / *N*_m_	28 / 22	29 / 25	.597
age, *M* ± *SD*	24.9 ± 3.9	24.7 ± 4.1	.899
laterality index, *M* ± *SD*	93.1 ± 12.1	95.1 ± 9	.493
external visual imagery, *M* ± *SD*	1.7 ± 0.6	2 ± 0.7	.079
internal visual imagery, *M* ± *SD*	1.6 ± 0.5	1.8 ± 0.7	.191
kinesthetic imagery, *M* ± *SD*	1.8 ± 0.8	1.8 ± 0.8	.255

**Table 2:** Design of the study including six test days.

Session	Session 1	Sessions 2–4	Session 5	Session 6
Experimenter	present	not present	present	present
Experimental phases	pretest		pretest	pretest
	practice		posttest	
	free generation		recognition	

## Data Availability

The data and material is provided in the link: https://osf.io/brd72

## Footnotes

1 The data from the posttests will be analyzed in another manuscript that focusses on consolidation mechanisms showing changes within each session and changes between sessions.
