# Supplementary material for: Time course of learning sequence representations in action imagery practice
Source: Hum Mov Sci. Author manuscript; Available in PMC 2023 Feb 4. (PMC7614144; doi:10.1016/j.humov.2022.103050)
Supplement: Appendix [file EMS163676-supplement-Appendix.pdf]

Appendix A

Statistical values of the ANOVA on RTs. Factors and factor levels were Practice hand (left, right), Practice group (mental practice, physical practice, control practice), Test hand (practice, transfer), Sequence (practice, mirror, different), and Session (1, 2, 3, 4, 5, 6).

	df1, df2	*F*	*p*	η_p^2
Practice hand	1, 81	0.2	.644	<.01
Practice hand x practice group	1, 81	1	.329	.01
Practice hand x test hand	1, 81	13.4	<.001	.14
Practice hand x session	2.7, 221.4	0.8	.737	.01
Practice hand x practice group x test hand	1, 81	0.2	.7	<.01
Practice hand x practice group x session	2.7, 221.4	0.4	.048	.03
Practice hand x test hand x session	4.6, 371	1	.422	.01
Practice hand x practice group x test hand x session	4.6, 371	1.5	.196	.02
Practice group	1, 81	<0.1	.906	<.01
Practice group x test hand	1, 81	<0.1	.954	<.01
Practice group x session	2.7, 221.4	0.8	.496	.01
Practice group x test hand x session	4.6, 371	1.1	.379	.01
Test hand	1, 81	0.1	.815	<.01
Test hand x session	4.6, 371	0.8	.509	.01
Session	2.7, 221.4	234.2	<.001	.74
Sequence	1.7, 139.4	53.9	<.001	.4
Sequence x practice hand	1.7, 139.4	0.3	.721	<.01
Sequence x practice group	1.7, 139.4	7.8	.001	.09
Sequence x test hand	1.8, 148.4	25.7	<.001	.24
Sequence x session	8.8, 716.5	8.7	<.001	.1
Sequence x practice hand x practice group	1.7, 139.4	1.2	.304	.01
Sequence x practice hand x test hand	1.8, 148.4	1.5	.217	.02
Sequence x practice hand x session	8.8, 716.5	2.1	.504	.01
Sequence x practice group x test hand	1.8, 148.4	4.9	.011	.06
sequence x practice group x session	8.8, 716.5	2.1	.028	.03
Sequence x test hand x session	8.9, 717.8	1.8	.073	.02
Sequence x practice hand x practice group x test hand	1.8, 148.4	1.6	.203	.02
Sequence x practice hand x practice group x session	8.8, 716.5	0.8	.577	.01
Sequence x practice group x test hand x session	8.9, 717.8	0.5	.862	.01
Sequence x practice hand x practice group x test hand x session	8.9, 717.8	0.4	.948	.01

Appendix A. Supplementary data

Supplementary data to this article can be found online at https://doi.org/10.1016/j.humov.2022.103050.
